# Indications and outcomes of second aortic procedures after acute type A dissection repair

**DOI:** 10.1093/icvts/ivae076

**Published:** 2024-04-30

**Authors:** Mohammed Morjan, Carlos-A Mestres, Vedran Savic, Mustafa Gerçek, Mathias Van Hemelrijck, Juri Sromicki, Omer Dzemali, Diana Reser

**Affiliations:** Department of Cardiac Surgery, University Hospital Zürich, Zürich, Switzerland; Department of Cardiovascular Surgery, Heinrich Heine University, Medical Faculty, Duesseldorf, Germany; Department of Cardiac Surgery, University Hospital Zürich, Zürich, Switzerland; Department of Cardiac Surgery, University Hospital Zürich, Zürich, Switzerland; Clinic for Cardiac Surgery and Pediatric Cardiac Surgery, Heart Center Duisburg, Duisburg, Germany; Department of Cardiac Surgery, University Hospital Zürich, Zürich, Switzerland; Department of Cardiac Surgery, University Hospital Zürich, Zürich, Switzerland; Department of Cardiac Surgery, University Hospital Zürich, Zürich, Switzerland; Department of Cardiac Surgery, University Hospital Zürich, Zürich, Switzerland; Herzklinik Hirslanden, Zürich, Switzerland

**Keywords:** Acute aortic dissection, Re-do surgery

## Abstract

**OBJECTIVES:**

Aortic arch or aortic root replacement is not performed in all cases of acute type A aortic dissection (ATAD), and a second aortic procedure will become necessary over time for some patients. Indications and outcomes, of second aortic procedures have not been studied extensively.

**METHODS:**

Characteristics and in-hospital outcomes of all patients undergoing surgical repair for type A acute aortic dissection were analysed and patients needing second aortic procedure during follow-up were identified. The latter group was divided in 2 subgroups: on-pump includes patients operated on using cardiopulmonary bypass and off-pump without cardiopulmonary bypass.

**RESULTS:**

A total of 638 patients underwent surgery for ATAD; 8% required a second aortic procedure. The most frequent indication for the second aortic procedure was dehiscence of suture lines (44%), followed by arch dilatation (24%). In-hospital mortality was 12%. Isolated ascending aorta replacement at the first surgery was associated with higher incidence of second aortic procedure (*P* = 0.006). Most patients in the on-pump group underwent a proximal reoperation (75%), with a mortality rate of 14.2%. In-hospital mortality of patients in the off-pump group was 7.7%. Long-term survival analysis showed no difference between groups (*P* = 0,526), Off-pump patients have greater likelihood of a second intervention during follow-up (*P*  = 0.004).

**CONCLUSIONS:**

Extended aortic root surgery and customized aortic arch repair in ATAD could be reasonable to reduce the incidence and mortality of high-risk second aortic procedures.

## Graphical abstract

**Figure:**
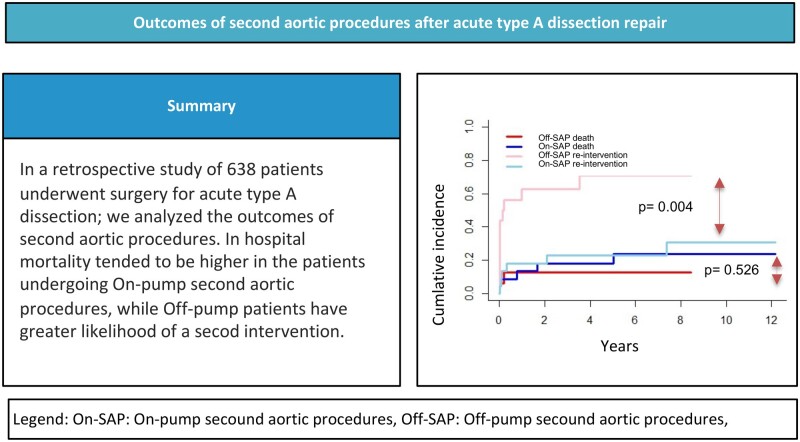

## INTRODUCTION

Acute type A aortic dissection (ATAD) is still associated with high morbidity and mortality despite improved outcomes of surgery, which remains the gold standard of therapy [1]. 

The primary aim of surgery in ATAD is to prevent intrapericardial aortic rupture; this is accomplished by excision of the proximal entry tear and replacement of the ascending aorta (RAA) eventually with total arch replacement (TAR) and/or aortic root replacement (ARR) [2]. Extension of surgery to ARR or TAR is related to anatomical and pathological factors and aims at treating coronary ostial dissection, correction of aortic valve insufficiency, restoration of true lumen flow and correction of distal malperfusion, when present [3]. TAR and/or ARR is often performed to decrease or avoid the risk of late reoperation due to progressive aortic dilatation or re-dissection. Regardless of the extension of surgical repair, pathologic aortic tissue is left *in situ*, and a proportion of successfully operated patients, will need a secondary aortic procedure (SAP) during their follow-up.

In this study, we analysed the characteristics and outcomes of SAP in patients operated primarily for ATAD and investigated whether the extension of the first surgery may predict the need for SAP.

## PATIENTS AND METHODS

### Ethical statement

The Ethics Committee/Institutional Review Board approved this retrospective study (File number 2017-00824, 5 June) and waived the need for informed written consent for all patients until 31 December 2015. To be allowed to add those operated until 31 December 2018, an amendment from the Ethics Committee was obtained (Amendment File number 2017-00824, Kantonal Ethikkommission Zürich 04.02.2019) and we retrospectively collected signed informed consent, either from the patients, relatives or next of kin.

### Study design

This is a retrospective single-centre study of all 638 patients who underwent emergency surgery for ATAD Between 1 January 2006 and 31 December 2018, at the department of cardiac surgery of the University Hospital of Zürich. For follow-up, patients were regularly seen at the outpatient clinic or were contacted by telephone. Patients needing SAP during the follow-up were identified until the closing date for the data analysis.

### Data collection

Baseline characteristics, operative variables and early in-hospital outcomes for all patients undergoing emergency surgical repair for ATAD were collected and patients needing SAP during the follow-up were identified. Baseline characteristics, operative variables, and postoperative outcomes of SAP group at the second procedure were collected and analysed. SAP group was divided in 2 subgroups: on-SAP group includes patients undergoing SAP using cardiopulmonary bypass (CPB) and off-SAP group includes those undergoing off-pump SAP such as thoracic endovascular aortic repair (TEVAR), debranching or transcatheter aortic valve implantation.

### Definitions

ATAD has been defined as per the 2014 European Society of Cardiology Guidelines on the diagnoses and treatment of aortic diseases [4]. Replacement of the ascending aorta was intended as ascending aorta replacement proximally to the innominate artery with or without open distal anastomosis. Hemiarch replacement (HAR) was defined as resection of the concavity of the aortic arch down to the proximal descending thoracic aorta without arch vessel reimplantation. Total Arch Replacement was intended as replacing the entire aortic arch from the offspring of the Innominate artery to a point beyond the offspring of the left subclavian artery (LSA) [5]. The latter 2 definitions have been incorporated as per the expert consensus document of the European Association for Cardio-Thoracic Surgery and the European Society for Vascular Surgery [6]. A proximal second aortic procedure was intended as any procedure at the level of the aortic root or proximal suture line, while a distal one was intended as any procedure at the level of the aortic arch or distal suture line.

### Surgical techniques

#### Primary surgical procedure

After median sternotomy and systemic heparinization, CPB was initiated usually with cannulation of right subclavian artery (RSA). If not feasible, femoral cannulation or direct cannulation of the aorta was performed. Crystalloid cardioplegia was used in most cases. Open distal anastomosis was performed under hypothermic circulatory arrest and selective antegrade cerebral perfusion. In addition to RAA, distal and proximal repair of the aorta were achieved using adhesive biologic tissue glue in most cases, if extended replacement was not performed. Single or double felt strips were often used to reinforce suture lines between the prosthetic vascular graft and the aortic wall as per the operating surgeon’s preference.

#### Secondary aortic procedure using cardiopulmonary bypass

A preoperative thoraco-abdominal computed tomographic (CT) scan was performed in all cases. Arterial cannulation site was selected based on anatomical characteristics in relation to the first surgery, and the planned surgical strategy. RSA remains the preferred cannulation site. However, LSA, femoral cannulation or direct cannulation of the aorta (or aortic graft) were used when necessary.

### Off-pump secondary aortic procedure (Off-SAP)

Patients considered at high risk for a procedure with CPB and suitable for endovascular techniques were treated with hybrid or endovascular procedures, including debranching of 1 or more supra-aortic vessel and TEVAR.

### Follow-up after the index operation

Patients operated on for ATAD received a thoraco-abdominal CT scan before discharge. After discharge patients were seen 1 month and 6 months after the operation, and yearly thereafter receiving a regular CT scan. In case of any abnormal finding, close serial CT scans were performed to evaluate any pathological progression.

### Indication for SAP

Decision for SAP was based on current available guidelines [5–7], the individual patient risk profile as well as the local experience. Generally, in case of aortic aneurysm, exceeding 55 or >10 mm aortic diameter increases per year, intervention was indicated. In case of asymptomatic dehiscence or pseudoaneurysm of a suture line, indication for SAP was confirmed if any progression was observed on serial CT scans.

### Follow-up after SAP

For follow-up after SAP, the patients were seen at the outpatient clinic or were contacted by telephone. The closing date for the analysis of this cohort was 20 February 2023.

### Statistics

All statistical analyses were performed using IBM SPSS Statistics Version 23 (IBM Corp; Armonk, New York, USA) and R 4.3.2 (R Foundation for Statistical Computing, Vienna, Austria). Data were explored for outliers and normality by using normal Q–Q plots. Categorical variables are presented as frequency with percentage, and continuous variables as mean with standard deviation (or median and IQR). To Compare all ATAD and all SAP, and On-SAP and off-SAP the Pearson's χ^2^ test (or Fisher’s exact test) was used to compare categorical variables and unpaired Student’s *t*-test (or Mann–Whitney *U*-test) was used to compare continuous variables. *P*-values <0.05 were considered statistically significant. Long-term survival, freedom from any reintervention and competing risk analysis were performed using Fine-Gray models.

In addition, univariable and multivariable Logistic regression models were constructed to find out a possible effect of surgical extension at the first surgery on SAP. The patients who died after the FAP were not included into the model. All variables were included in the univariable analysis. Variables with a *P*-value of <0.2 were included in the multivariable analysis.

## RESULTS

### Primary surgery

#### Patient characteristics

Between 1 January 2006 and 31 December 2018, a total of 638 patients underwent emergency surgery for ATAD (group A). Over a mean follow-up of 54 ± 36 months, a total of 504 patients were regularly seen at the outpatient clinic. Forty-one patients (8%) required SAP.

Baseline characteristics of the whole ATAD cohort and the SAP group are summarized in Table 1.

**Table 1:** Baseline characteristics at primary surgery

Variable	All ATAD	SAP
*N*	597	41
Age (years)	64 (13)	58 (12)
Female	185 (31%)	10 (24%)
Male	412 (69%)	31 (76%)
Hypertension	428 (67%)	25 (61%)
EuroSCORE II (%)	16 (13)	11 (5)
Diabetes mellitus	25 (4%)	0 (0%)
Hypercholesterolaemia	137 (23%)	9 (22%)
COPD	31 (5%)	3 (7%)
Renal failure	34 (5%)	2 (4.9%)

Mean with standard deviation or number of patients with percentage.

ATAD: acute type A aortic dissection; COPD: chronic obstructive pulmonary disease; SAP: secondary aortic procedure.

#### Intraoperative data at the index operation

The RSA was cannulated in 39 patients of the SAP group (95%), in 2 cases (5%) the aorta was directly cannulated. In 14 patients (34%) in the SAP group, surgery was limited to isolated RAA and in 15% in the whole cohort, intraoperative data of the whole cohort and the SAP group by the first surgery are summarized in Table 2.

**Table 2:** Intraoperative data at primary surgery

Variable	All ATAD	SAP
Cardiopulmonary bypass time (min)	200 (90)	178 (79)
Aortic cross-clamping time (min)	102 (59)	84 (47)
Cerebral perfusion time (min)	27 (25)	23 (22)
Hypothermia (°C)	27 (6)	27 (8)
Isolated ascending aorta replacement	99 (15%)	14 (34%)
Aortic root repair	110 (17%)	8 (19%)
Aortic root replacement	199 (31%)	10 (24%)
Mechanical valve	107 (17%)	6 (15%)
Biological valve	143 (23%)	7 (17%)
Hemiarch replacement	331 (52%)	18 (44%)
Complete arch replacement	100 (15.7%)	3 (7%)

Mean with standard deviation or number of patients with percentage.

ATAD: acute type A aortic dissection; SAP: secondary aortic procedure.

#### Morbidity and mortality

No differences in postoperative morbidity were noted. Outcomes of the first surgery are summarized in Table 3.

**Table 3:** In-hospital outcomes at primary surgery

Variable	All ATAD	SAP group	OR	*P*-value
In-hospital mortality	112 (17,5%)	0 (0%)	0.2	**0.01**
ECMO/ECLS	30 (4.7%)	0 (0%)	0.9	0.09
Neurological event	97 (15.2%)	5 (12.4%)	0.8	0.2
Pneumonia	143 (22.4%)	10 (24%)	1.1	0.7
Renal impairment (creatinine >200 μmol/l)	190 (29.8%)	17 (42%)	1.2	0.5
Myocardial infarction	34 (5.3%)	0 (0%)	0.4	0.3

Mean with standard deviation or number of patients with percentage.

ATAD: acute type A aortic dissection; OR: odds ratio; SAP: secondary aortic procedure; ECMO: extracorporeal membrane oxygenation; ECLS: extracorporeal life support.

Bold represents statistically significant value.

#### Multivariable logistic regression

Isolated RAA, TAR and ARR at the index operation were found to have a *P*-value of <0.2 at the univariable logistic regression models and were inserted in the multivariable model. Isolated RAA was significantly associated with the need of SAP during the follow-up (*P*  = 0.006, Table 4).

**Table 4:** Multivariable logistic regression analysis

Variable	Odds ratio	95% CI	*P*-value
Isolated ascending aorta replacement	2.886	1.355–6.144	0.006
Total arch replacement	0.531	0.156–1.805	0.31
Aortic root replacement	1.045	0.467–2.338	0.914

CI: confidence interval.

### Second aortic procedure

#### Patient characteristics and indication

Most SAPs were elective (*N* = 40, 96%) and 1 case was defined as urgent. The median time to reintervention was 6 years [2–10] The mean EuroSCORE II was 18 ± 15. The most frequent indication for the SAP was the dehiscence of 1 or more suture lines (44%), followed by progressive arch dilatation (24%). Baseline characteristics of the second procedure and indications for SAP are summarized in Table 5.

**Table 5:** Preoperative characteristics, secondary aortic procedure group

Variable	All SAP (*N* 41)	ON-SAP (*N* 28)	Off-SAP (*N* 13)	OR	*P*-value
Age (years)	60 (11)	59 (11)	58 (11)		0.34
EuroSCORE II (%)	18 (15)	18 (15)	18 (5)		0.55
Indication for SAP					
Progressive arch aneurysm	10 (24%)	3 (11%)	7 (54%)	0.1	<0.01
Progressive root aneurysm	3 (7%)	3 (11%)	0 (0%)	0.6	0.25
Aortic valve regurgitation	6 (15%)	6 (21%)	0 (0%)	0.6	0.09
Endocarditis/graft infection	3 (7%)	3 (11%)	0 (0%)	0.6	0.25
Dehiscence or proximal suture line pseudoaneurysm	9 (22%)	9 (32%)	0 (0%)	0.6	0.60
Dehiscence or distal suture line pseudoaneurysm	8 (20%)	3 (11%)	5 (38%)	0.2	<0.01
Re-dissection aortic arch	1 (2%)	0 (0%)	1 (8%)	0.3	0.52
Re-dissection aortic root	1 (2%)	1 (6%)	0 (0%)	0.7	0.52

Mean with standard deviation or number of patients with percentage.

Off-SAP: off-pump secondary aortic procedure; On-SAP: secondary aortic procedure using cardiopulmonary bypass; OR: odds ratio; SAP: secondary aortic procedure.

#### Intraoperative data

In 21 patients (51%), a proximal reoperation was needed; in 16 patients (39%), a distal procedure was performed, and in 4 patients (9.7%), a combination of proximal and distal procedure was performed. Intraoperative data and details of reinterventions are reported in Supplementary Material, Table S1.

#### On-SAP

CPB was needed in 28 patients (68% of the entire SAP cohort). The RSA was cannulated in 10 of them (36%), LSA in 5 (18%), the aorta (or aortic graft) was directly cannulated in 3 patients (11%), and the femoral artery was preferred in 4 cases (14%). In 6 cases (21%), a combination of subclavian and femoral arterial cannulation was used.

#### Off-SAP

Thirteen patients (32%) underwent off-pump endovascular procedures including debranching of 1 or more supra-aortic vessel combined with TEVAR. In 1 patient, an additional transcatheter aortic valve implantation was performed due to severe aortic regurgitation.

#### Morbidity and mortality

In-hospital mortality for SAP patients was 12% (5 patients). When CPB was required, mortality was 14.3%. Two patients presented with post-operative low cardiac output syndrome and multiorgan failure; 1 patient had extensive neurological damage and 1 patient had unexplained fulminant disseminated intravascular coagulation. Three patients who died underwent a proximal SAP (75%); 1 had a combined proximal and distal SAP. Postoperative morbidity and mortality are presented in Supplementary Material, Table S2. One in-hospital mortality was registered in a patient who underwent endovascular procedures (off-SAP) due to intracranial bleeding.

#### Follow-up after SAP

**Figure 1:**
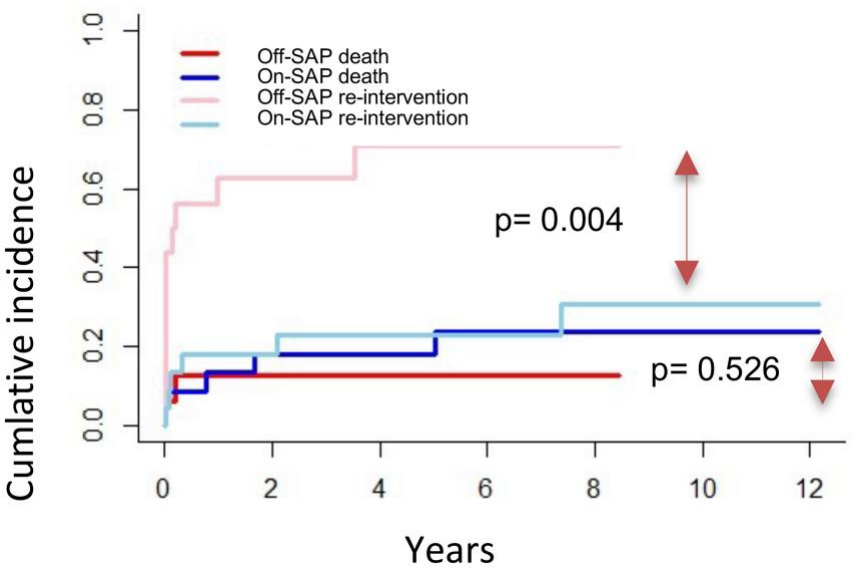
Fine–Gray models for mortality and second aortic procedure.

The median follow-up of this group was 6 years [2–10] which was 95% complete. Considering mortality as a competing event with SAP, Fine–Gray models were utilized. No significant difference in mortality was found between on-pump SAP and off-pump SAP patients (*P* = 0.526, Fig. 1). 84% of patients in the off-SAP group underwent at least 1 reintervention during the follow-up, including extension or correction of TEVAR, new debranching or new implantation of stent in 1 or more vessels, compared to 25% in the On-SAP group, including TEVAR in the majority of cases (*P*  = 0.004, Fig. 1).

## DISCUSSION

In this cohort, there is a low incidence of SAP when compared to other series reported in literature. When only On-SAP reoperations were considered, the incidence was even lower (5.5%).

Geirsson *et al.* [8] reported an incidence of 11% in their study and all patients were treated by open surgery. Pugliese *et al.* [9] reported an incidence of 13% in a series of 141 patients who survived a first ATAD repair. A similar incidence was reported from Concistrè *et al.* [10], whereas a more recent sub-study from the Nordic Consortium for Acute Type A Aortic Dissection reported an incidence rate similar to ours [11]. The authors of this latter study attributed the low incidence to the improved outcomes of ATAD surgery in the last years. We see an additional explanation in the close and accurate clinical and radiological follow-up that allows us to observe the progression of any abnormal postoperative finding and to maintain a safe conservative approach in case of asymptomatic findings without radiological progression.

In the present study, the most frequent indication for SAP was the dehiscence of 1 or more suture lines. This is an infrequent but a well-known complication of aortic surgery. The causative mechanisms are not clearly elucidated. However, factors like a local inflammatory reaction, slovenly surgical technique and tissue fragility could play a role; the use of tissue glue was not associated with suture failure [12].

In the SAP group, the frequency of isolated RAA at the first surgery was high. It can be speculated that limiting surgery to the ascending aorta could increase the incidence of SAP, This was confirmed by multivariable logistic regression which identified isolated RAA as a risk factor for SAP. On the other hand, the extent of surgery to the hemiarch and TAR was low in the SAP group at the first operation. The multivariable logistic regression failed to show a significant relationship. Nonetheless, it should be recognized that surgery extension to hemiarch, where arch vessel reimplantation is not needed, could be easily undertaken in setting of ATAD surgery without additional risks related to a longer time under total circulatory arrest as in case of TAR. This consideration is consistent with the work of Kim *et al.* [13], who found that TAR was associated with higher morbidity and mortality compared with HAR, with no significance difference in SAP rates between the 2 surgical strategies.

A more conservative approach intended as less TAR could be adopted to reduce surgical mortality and morbidity in acute setting. Rylski *et al.* [14] found that HAR can be performed with low mortality and low aortic arch reoperation rate. In addition, in the present study, most of the distal SAP were performed with endovascular techniques with low mortality. Endovascular interventions for distal aortic repair after ATAD surgical repair were associated with lower in-hospital mortality and better survival as reported from another group [15]. Nonetheless, the rate of re-interventions during the follow-up in our cohort was high. On one hand, the extension of surgery to the hemiarch in ATAD remains reasonable to reduce surgical mortality. On the other hand, the need for multiple interventions should be taken into consideration and a case-by-case decision based on the clinical presentation at the first surgery and the surgeon’s individual experience remains essential.

Different groups advocated a minimalistic proximal aortic approach in ATAD defined as root repair, using tissue glue or similar conservative techniques, rather than replacement. Dell'Aquila *et al.* [16] reported a series of 319 consecutive patients who underwent RAA with preservation of the aortic root. The incidence of SAP was 10.8% with a mortality of 16.7%. The authors argued that a minimalistic root approach was justified due to the technical simplicity and reproducibility with acceptable rate of SAP and mortality. However, the authors' conclusions were weighed against a relatively high mortality in primary procedures (34%). In a multi-institutional report [17], the same authors reported early outcomes in 117 aortic reoperations after an initial operation for ATAD. The rate of proximal SAP was 66.6% with a mortality of 14.1%, which was considered acceptable to define the minimalistic root approach as preferable.

In a propensity score-matched analysis, Castrovinci *et al.* [18] compared outcomes of ARR versus conservative root management in ATAD. Conservative and aggressive root management were found to provide similar results for early and late mortality. However, freedom from proximal aortic reintervention was high as 96% in patients who underwent aggressive root replacement. Similar data were reported by Di Eusanio *et al.* [19] from The International Registry on Aortic Dissection, where a more aggressive root management was encouraged, especially in young patients and patients with connective tissue diseases.

Although no difference was noted between root repair and root replacement rates at primary surgery in the present cohort, most patients needing SAP underwent a proximal reoperation (75%), with a mortality rate like that of the entire SAP group (14.2%).

Overall mortality in the SAP group remains acceptable (9.7%) when compared to the other series [20]. However, all patients who died had a on-SAP (14.2%). This represents a higher mortality when compared to the total group. Based on this finding, we suggest considering the high risk of late aortic root reoperation when opting for a surgical approach limited to ascending aortic replacement and advocate a more radical approach of the aortic root. This could further reduce the rate of high-risk late SAP after ATAD repair.

### Limitations

The limitations of our study are the following: it is single centre, retrospective and observational. Furthermore, the number of patients needing SAP is small in comparison to the whole cohort. Furthermore, the definition of extension of surgery to aortic root and aortic arch was reported as described in surgical reports, which could be influenced by the surgeon’s interpretation of dissection extension. In addition, data regarding patients with a connective tissue disease were not available in our database. Although the patients are followed at our clinic, we cannot exclude that a given patient might have been followed at another centre without retrieving accurate follow-up information.

## CONCLUSION

SAP is associated with high mortality and morbidity, especially if an open aortic surgery is needed. Off-pump SAP is associated with a higher probability of further interventions. A more aggressive surgical approach on the aortic root and a customized approach at the aortic arch in ATAD could be a reasonable option aiming at reducing the incidence and mortality of high-risk second aortic procedures after ATAD repair.

## Supplementary Material

ivae076_Supplementary_Data

## Data Availability

The data underlying this article are available in the article and in its online supplementary material.

## Notes

Presented at the 52 Annual Meeting of the German Society of Thoracic and Cardiovascular Surgery (GSTCVS), Hamburg, Germany, 11–14 February 2023,

## ABBREVIATIONS

ATAD: Acute type A aortic dissection
ARR: Aortic root replacement
CPB: Cardiopulmonary bypass
CT: Computed tomography
HAR: Hemiarch replacement
IRAD: The International Registry on Aortic Dissection
LSA: Left subclavian artery
RAA: Replacement of the ascending aorta
RSA: Right subclavian artery
SAP: Secondary aortic procedure
TAR: Total arch replacement
TEVAR: Thoracic endovascular aortic repai
