# Supplementary material for: Indications and outcomes of second aortic procedures after acute type A dissection repair
Source: Interdiscip Cardiovasc Thorac Surg. 2024 Apr 30;38(5):ivae076. doi: 10.1093/icvts/ivae076 (PMC11090986; doi:10.1093/icvts/ivae076)
Supplement: ivae076_Supplementary_Data [file ivae076_supplementary_data.zip › Supplemental table 2.docx]

Supplemental table 2 - In hospital outcomes, SAP group:

| **Variable** | **ON-SAP (N 28)** | **Off-SAP (N 13)** | **OR** | **p-value** |
| --- | --- | --- | --- | --- |
| **In-hospital mortality** | 4 (9.7%) | 1(7.7%) | 2.1 | 0.51 |
| **ECMO/ECLS** | 3(11%) | 0 | 0.6 | 0.44 |
| **Neurological event** | 3(11%) | 2(15.4%) | 0.7 | 0.57 |
| **Pneumonia** | 3(11%) | 2(15.4%) | 0.7 | 0.93 |
| **Renal impairment (creatinine >200micromol/L)** | 3(11%) | 1(7.7%) | 1.5 | 0.83 |
| **Myocardial infarction** | 3(11%) | 0 | 0.6 | 0.51 |

Mean with standard deviation or number of patients with percentage. **ATAD:** Acute type A aortic dissection **SAP:** Secondary aortic procedure. **ECMO**: extracorporeal membrane oxygenation. **ECLS:** ExtraCorporeal Life Support
