# Supplementary material for: Indications and outcomes of second aortic procedures after acute type A dissection repair
Source: Interdiscip Cardiovasc Thorac Surg. 2024 Apr 30;38(5):ivae076. doi: 10.1093/icvts/ivae076 (PMC11090986; doi:10.1093/icvts/ivae076)
Supplement: ivae076_Supplementary_Data [file ivae076_supplementary_data.zip › Supplemental table 1.docx]

Supplemental table 1 – Intraoperative Data, SAP group :

| **Variable** | **ON-SAP**  **(N28)** | **Off-SAP**  **(N13)** |
| --- | --- | --- |
| Need of cardiopulmonary bypass time | 28 (68 %) | - |
| Cardiopulmonary bypass time [min] | 224 (97) | - |
| Aortic cross-clamping time [min] | 111 (67) | - |
| FET | 2(4.9%) | - |
| David OP | 2(4.9%) | - |
| Arch Replacement | 1 (2.4%) | - |
| Bentall | 12(29%) | - |
| Bentall+Arch replacement | 1(2.4%) | - |
| Ascending + Arch | 3(7.3%) | - |
| Re ascending replacement | 5(12%) | - |
| TEVAR ± Aortic debranching | - | 12 (29%) |
| Aortic debranching + TEVAR + TAVI | - | 1(2.4%) |
| Suture line repair (proximal) | 2 (4.9%) | - |

Mean with standard deviation or number of patients with percentage. **On-SAP:** Secondary aortic procedure using CPB**, Off-SAP:** Off pump Secondary aortic procedure. **FET:** frozen elephant trunk. **TEVAR:** Thoracic endovascular aortic repair. **TAVI**: transcatheter aortic valve implantation
